# Soft Capsule Magnetic Millirobots for Region-Specific Drug Delivery in the Central Nervous System

**DOI:** 10.3389/frobt.2021.702566

**Published:** 2021-07-22

**Authors:** Lamar O. Mair, Georges Adam, Sagar Chowdhury, Aaron Davis, Dian R. Arifin, Fair M. Vassoler, Herbert H. Engelhard, Jinxing Li, Xinyao Tang, Irving N. Weinberg, Emily E. Evans, Jeff W.M. Bulte, David J. Cappelleri

**Affiliations:** ^1^ Weinberg Medical Physics, Inc., North Bethesda, MD, United States; ^2^ Multi-Scale Robotics and Automation Lab, School of Mechanical Engineering, Purdue University, West Lafayette, IN, United States; ^3^ Russel H. Morgan Department of Radiology and Radiological Science, Division of Magnetic Resonance Research, The Johns Hopkins University School of Medicine, Baltimore, MD, United States; ^4^ Cellular Imaging Section and Vascular Biology Program, Institute for Cell Engineering, The Johns Hopkins University School of Medicine, Baltimore, MD, United States; ^5^ Cummings School of Veterinary Medicine, Tufts University, North Grafton, MA, United States; ^6^ Affiliated Neurosurgery Corporation, Chicago, IL, United States; ^7^ Department of Bioengineering, University of Illinois at Chicago, Chicago, IL, United States; ^8^ Department of Biomedical Engineering, Institute for Quantitative Health Science and Engineering, Michigan State University, East Lansing, MI, United States; ^9^ Department of Physics, Elon University, Elon, NC, United States; ^10^ Departments of Oncology, Biomedical Engineering and Chemical and Biomolecular Engineering, The Johns Hopkins University School of Medicine, Baltimore, MD, United States; ^11^ Weldon School of Biomedical Engineering, Purdue University, West Lafayette, IN, United States

**Keywords:** magnetic millirobot, rotating magnetic fields, alginate microcapsule, drug delivery, surface walkers

## Abstract

Small soft robotic systems are being explored for myriad applications in medicine. Specifically, magnetically actuated microrobots capable of remote manipulation hold significant potential for the targeted delivery of therapeutics and biologicals. Much of previous efforts on microrobotics have been dedicated to locomotion in aqueous environments and hard surfaces. However, our human bodies are made of dense biological tissues, requiring researchers to develop new microrobotics that can locomote atop tissue surfaces. Tumbling microrobots are a sub-category of these devices capable of walking on surfaces guided by rotating magnetic fields. Using microrobots to deliver payloads to specific regions of sensitive tissues is a primary goal of medical microrobots. Central nervous system (CNS) tissues are a prime candidate given their delicate structure and highly region-specific function. Here we demonstrate surface walking of soft alginate capsules capable of moving on top of a rat cortex and mouse spinal cord *ex vivo*, demonstrating multi-location small molecule delivery to up to six different locations on each type of tissue with high spatial specificity. The softness of alginate gel prevents injuries that may arise from friction with CNS tissues during millirobot locomotion. Development of this technology may be useful in clinical and preclinical applications such as drug delivery, neural stimulation, and diagnostic imaging.

## Introduction

Therapies loaded into milli- and microrobotic devices guided by optical, acoustic, electrophoretic, and magnetic forces have demonstrated potential for performing microsurgery (Xi et al., 2013), delivering payloads (Li et al., 2017), performing local biosensing functions (McNaughton et al., 2007), performing magnetic manipulations (Wang et al., 2021), sensing forces (Jing et al., 2019), and operating as magnetic resonance imaging contrast agents (Zabow et al., 2008, 2015). Clinical magnetic steering devices are currently in use, with others under development (Chautems et al., 2017). Particularly promising are efforts to use magnetic fields to guide synthetic milli- and microrobots to specific target locations in or on tissues (Sitti et al., 2015). Magnetic guidance is particularly advantageous for accomplishing such tasks as magnetic fields are minimally attenuated by tissues and are generally well tolerated across a wide range of field strengths and frequencies (Abbott et al., 2020). Using magnetic fields to direct the delivery of cellular, molecular, and protein therapies to specific regions of the body has the potential to increase therapeutic efficacy via targeted delivery and enhanced retention, while decreasing side effects by minimizing off-target deposition and tissue absorption. Efforts to understand microrobot transport *in vitro* and *in vivo* have resulted in an impressive array of nano- and microscale robot-like particles and devices for operation in and on tissues (Cao et al., 2019; Zhang et al., 2021). Advancing the capabilities for delivering payloads through and on top of tissues is a critical aspect of engineering particles and processes for payload delivery. Additionally, new particles and manipulation techniques have the potential to enable new methods of delivery with the possibility for improved therapeutic outcomes.

Surface walking or tumbling particles and assemblies whose translation relies on rotation near a solid-liquid boundary have been used for moving objects on smooth (Zhang et al., 2010) and rough synthetic surfaces (Bi et al., 2018), biofilm-laden surfaces (Mair et al., 2017), for generating fluid flows (Sing et al., 2010), for moving against flow (Karle et al., 2010), and for purifying proteins (Tung et al., 2014). However, the capabilities of these rotationally operated particles as drug delivery devices have been less studied despite their significant potential as targeted delivery tools. As one major objective for medical microrobots is the controlled delivery of payloads to target tissues, rotationally manipulated magnetic microrobots may offer a route for targeting therapies to the surfaces of accessible tissues or organs.

Nowhere is spatially controlled delivery more critical and more desirable than for the heterogenous central nervous system (CNS) tissue. Localizing drug treatments to these tissues could have major implications for treating neurological conditions ranging from psychiatric disorders to neurodegenerative diseases. Specifically, targeted delivery may allow the overall dose required to be reduced, thereby alleviating side effects and decreasing systemic toxicity. Ideally, medical microrobots would enable the physician or service provider to target a region of the CNS with minimal invasiveness, and with localized, concentration-tunable doses of therapeutics which would be absorbed by the treated areas while sparing untargeted tissues. The cortex and the spinal cord are potential targets for therapeutic delivery using magnetically guided microrobots. Both tissues are bathed in low viscosity cerebrospinal fluid, meaning that treatment of the tissues does not necessarily require microrobots to traverse dense tissues (Engelhard et al., 2020). Additionally, these tissues are not deep inside the body, making it easier to apply strong magnetic fields sufficient for manipulation. Thus, delivering therapeutics to the surfaces of the brain and to the spinal cord is amenable to particles too large to travel through dense tissues. Similarly, the hydrodynamics of the no-slip boundary commonly employed to enable directed transport of magnetic objects via rotation is a possible approach to guiding magnetic agents. The space between the cortex and the dura, and similarly the space surrounding the spinal cord, are sufficiently large to allow for microrobot operation, thereby eliminating numerous size and force constraints on the particle which are necessary for tissue-penetrating particles. Moreover, the soft alginate gel enclosing the millirobots prevents injuries that may arise from friction with fragile CNS tissues during locomotion of these millirobots.

Here we demonstrate soft, magnetic, millimeter-scale capsules capable of climbing steep inclines and translating against flows, as well as a proof-of-principle ability to “paint the CNS” with targeted delivery of brilliant green (BG) dye as a model small molecule. These experiments, performed using magnetically aligned nanorods in alginate capsules (MANiACs) (Mair et al., 2019), demonstrate the ability to paint the cortex and the spinal cord *ex vivo* by sequential run and pause manipulation of MANiACs using rotating magnetic fields strengths ≤40 mT. We demonstrate here that MANiACs can climb inclines as steep as 45° and validate that MANiACs can advance against biologically relevant fluid flow velocities. Overall, this work represents a first application of MANiACs for targeted *ex vivo* delivery of drug payload to selected tissue regions, and a first demonstration of surface tumblers capable of painting CNS tissues.

## Materials and Methods

### MANiAC Synthesis and Loading

**FIGURE 1:**
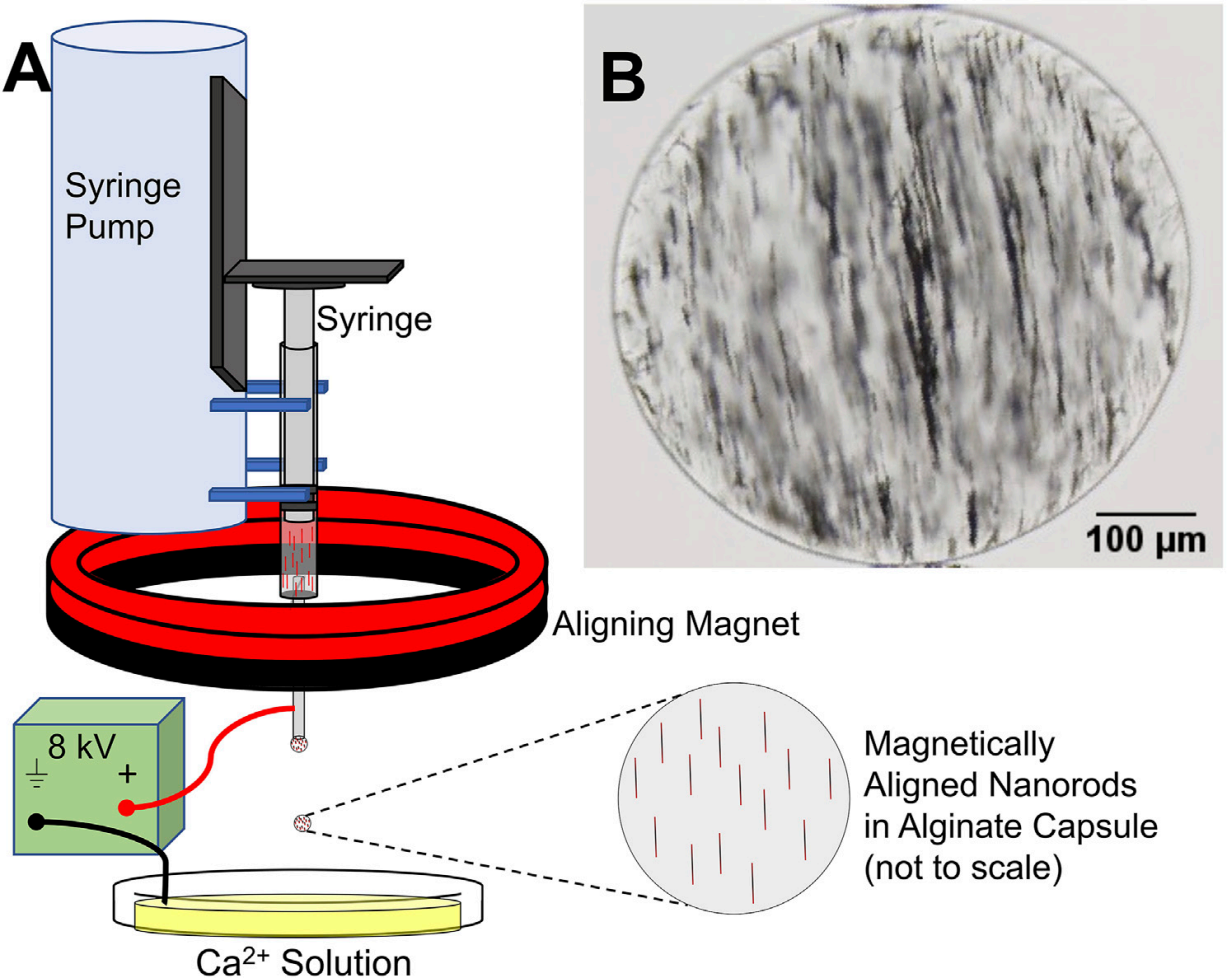
MANiAC synthesis. **(A)** Syringe with pre-aligned nanorods in liquid alginate is loaded into syringe pump and extracted at a rate of 100 μl/min. **(B)** MANiAC image.

MANiACs were synthesized by loading a syringe with a mixture of alginate and electrochemically grown nickel nanorods (NiNRs), pre-aligning the nanorods in the syringe with a permanent magnet, then extracting NiNR-loaded alginate droplets using a high voltage (∼8 kV) droplet generation method (Figure 1). Droplets were formed in the presence of an aligning magnetic field, ensuring that NiNRs remained aligned as they moved from the syringe tip and into the gelling calcium chloride solution. Capsules were generated at a rate of ∼4 Hz. Following synthesis, capsules were stored in a KRH buffer composed of 2.5 mM CaCl_2_, 194 mM NaOH, 4.7 mM KCl, 1.2 mM KH_2_PO_4_, 1.2 mM MgSO_4_∙7H_2_O, and 25 mM HEPES at pH = 7.4. Further details on alginate capsule formation can be found in previous reports (Barnett et al., 2011).

### *In Vitro* Manipulations: Translation up Inclines and Against Flow

Manipulations were performed using the MagnebotiX MFG-100 system (Magnebotix AG, Zurich, Switzerland, magnebotix.com) (Schuerle et al., 2013). The MFG-100 system incorporates eight electromagnets located underneath the sample workspace, each coil carrying a maximum of 20 A. While the MagnebotiX system is capable of supplying both magnetic gradients and gradient-free rotating fields, we utilize only the gradient-free uniform rotating field functionality of the system. Uniform fields between 10 and 40 mT were applied at rotation frequencies between 0.5 and 5 Hz.

**FIGURE 2:**
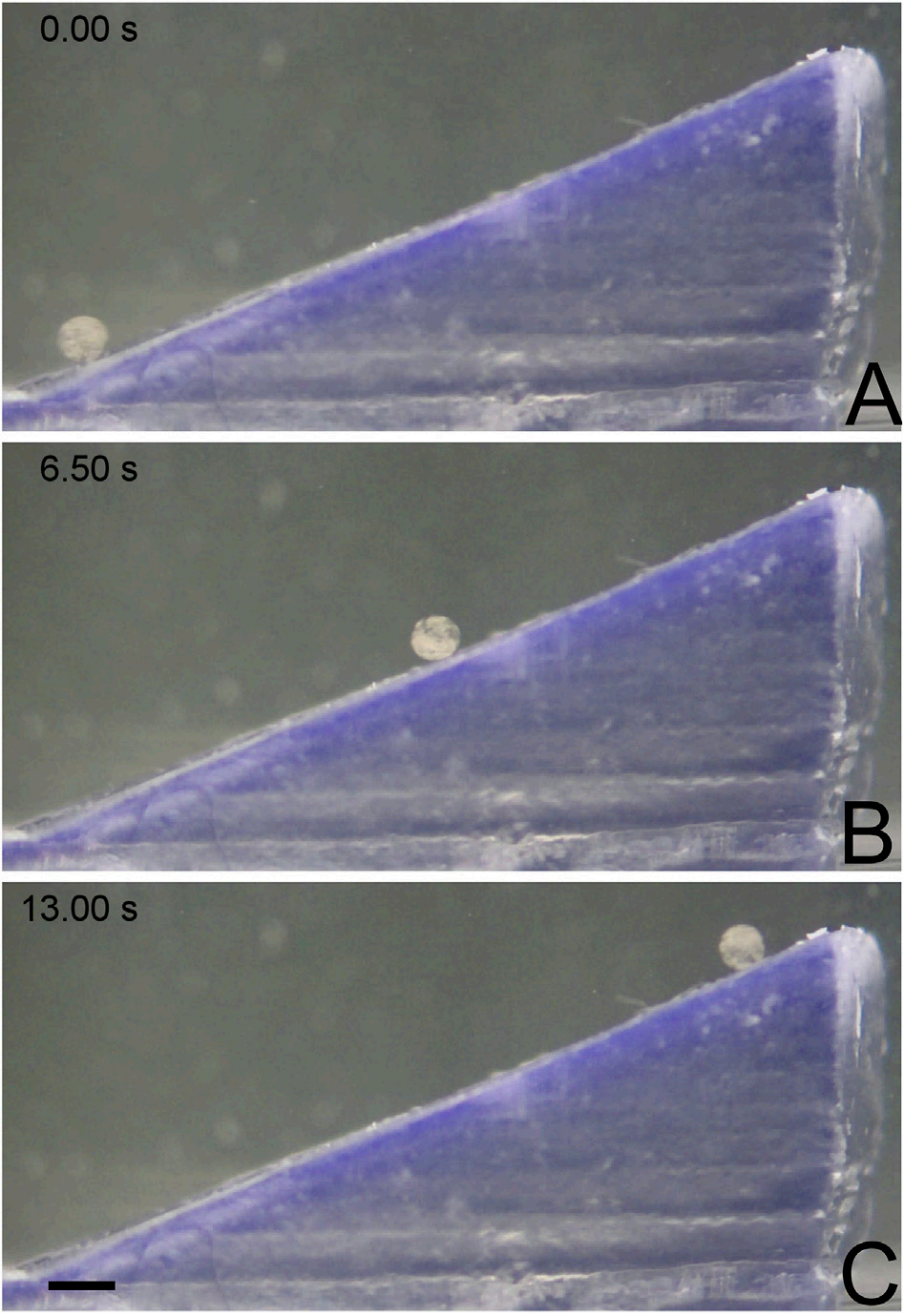
MANiAC climbing up a 25° incline. **(A)** MANiAC (lower left) starts at the bottom of the incline and climbs **(B)** to the top **(C)** in 13 s. Scale bar is 0.5 mm. Video included in Supplementary Information.

To better understand how MANiAC tumbling is affected by sloped edges, MANiACs were rotated to climb inclines ranging from 5 to 45° angles. Inclines were made on a Formlabs Form 1 printer with clear resin and a z-step height of 25 µm. Tests were performed by submerging the printed inclines in a petri dish filled with artificial cerebrospinal fluid (aCSF, Harvard Apparatus). Artificial CSF contained 150 mM sodium, 3.0 mM potassium, 1.4 mM calcium, 0.8 mM magnesium, 1.0 mM phosphate, and 155 mM chloride. Purity was measured at 99.5%, with a pH = 7.22, and an osmolarity of 281 mOsm/L. Prior to use, aCSF was filtered through a 0.22 μm filter. Density of aCSF is typically in the range of 1.008 mg/ml and viscosity is typically 1.01 mPa∙s (Miranda-Martínez et al., 2021). MANiACs were then placed at the base of the inclines using a micropipette. Climbing experiments were performed in 14 mT fields rotated at 2 and 5 Hz (Figure 2). Video data was collected from above and from the side and analyzed using ImageJ and Video Spot Tracker (free, https://cismm.web.unc.edu/software/).

To better understand how MANiAC motion would be affected by fluid flow, capsules were placed in fluidic channels under flowing fluid conditions. Controlled flow rates generated in a poly (dimethylsiloxane) (PDMS, Sylgard 184) channel were imposed on MANiACs being rotated at 1, 3, and 5 Hz. The PDMS channel was created by printing the shape of the channel out of 40% (w/v) Pluronic F-127 (as a sacrificial bioink) on a glass slide using a Cellink BioX. Then, PDMS was deposited around the printed shape and crosslinked at room temperature for 24 h, after which the channel was washed with cold water and a brush. Chilled water and brushing are used to remove the Pluronic sacrificial layer, which becomes liquid below 15°C. In order to create a controlled flow rate, a New Era NE-1000 syringe pump was connected to the fluidic channel and fluids were directly introduced into the channel. MANiACs were placed in the channel and magnetically rotated so as to move against the imposed flow. A magnetic field of 20 mT was used for all experiments and velocities were calculated and averaged across experiments (Figure 4). Experiments were performed in phosphate buffered saline (PBS) as well as 1, 5, and 10% disodium ethylenediaminetetraacetic acid (K2EDTA)-treated whole mouse blood diluted in PBS.

### Painting the CNS Experiments

Prior to CNS tissue painting experiments, alginate capsules were osmotically loaded with brilliant green dye (Sigma Aldrich) by immersion in 0.1 mg/ml BG solution in PBS. Two hours immersion time was sufficient to fully load capsules with the BG dye. After loading, capsules were rinsed and stored in DI water until experiments commenced, as the release of BG in DI water is minimal due to lack of osmotic pressure differences, as previously demonstrated (Mair et al., 2019).

Excised brain tissues were obtained from adult Sprague Dawley rats (BioIVT). Brains were placed in a 35 mm Petri dish and submerged in aCSF. After loading with BG, MANiACs were deposited onto the rat brain cortex and rotating fields were applied to induce translation. Rotating fields between 0.5 and 2 Hz at 20 mT were used for manipulation. Videos were collected from above the brain at 30 frames per second using a color charge-coupled device (CCD) camera (Basler puA1600-60uc, Basler AG, Ahrensburg, Germany). Manipulations included run-and-pause style dosing of BG onto the surface of the rat brain.

Similarly, for mouse spinal cord experiments, excised spinal cords were obtained from BABL/C mice (BioIVT), placed in a 35 mm Petri dish, and submerged in aCSF. For painting the spinal cord, a single MANiAC was loaded into the anterior median fissure of the mouse spinal cord. Magnetic fields of 20 mT were applied at frequencies between 0.5 and 2 Hz, with MANiACs moving for several seconds at a time, followed by pausing motion for 1.5 min. or more to allow for BG deposition into the tissue.

## Results

Our new MANiAC synthesis protocol demonstrated increased nanorod loading, improved shape uniformity, and improved overall capsule-to-capsule shape homogeneity as compared with our previous work. Improved shape and size homogeneity were attributed to implementation of more aggressive mixing techniques prior to capsule formation, and optimization of syringe tip position with respect to the calcium chloride solution anode. MANiACs were ∼380 ± 55 µm (mean standard deviation, *n* = 24). The NiNRs were observed to be evenly distributed within the alginate capsules although minor aggregation of the nanorods was unavoidable. Figure 1A outlines the MANiACs synthesis process and Figure 1B shows aligned nanorods within a capsule. All MANiACs demonstrated ferromagnetic ordering with pre-aligned magnetic orientation and were manipulatable using rotating magnetic fields of 0.5–5 Hz and 10–40 mT supplied by the MFG-100 system.

### MANiACs Climbing Inclines

As many surfaces of CNS tissues are inherently curved, micro- and millimeter scale soft robots designed to deliver therapeutics to specific locations must be able to move effectively over these curved and undulating surfaces. Experiments in climbing inclines were motivated by a need to understand what maximum inclines would be climbable with MANiACs under low frequency (<10 Hz) and low magnetic field (≤40 mT) conditions.

On a flat, open surface, rods exposed to a rotating magnetic field will experience torque described by

$$\tau_{m}=V_{m}\vec{M}\times \vec{B}=\pi r^{2}L\left|M_{r}\right|\left|B\right|\sin\theta$$

The applied magnetic torque ( $\tau_{m}$ ) can be calculated using the volume of magnetic material ( $V_{m}$ ), the magnetization of the rods ( $\vec{M}$ ), and the applied magnetic field $\left(\vec{B}\right)$ , or via knowledge of the remnant magnetization ( $M_{r}$ ), the rod radius (*r*), the length of the rod (*L*), and the lag angle between the nanorods and the applied magnetic field ( θ ) (Keshoju et al., 2007). Here we use the approximation that the rod’s magnetization is along the rod length and that the rotating field is sufficiently slow such that the lag angle θ is small. In ensemble, collections of these rods embedded in an alginate capsule transfer magnetic torque to the capsule as a whole, inducing rotation and, near a surface, translation (Mair et al., 2019). Here we focus on experiments which demonstrate climbing inclines thrice as steep as we originally demonstrated.

**FIGURE 3:**
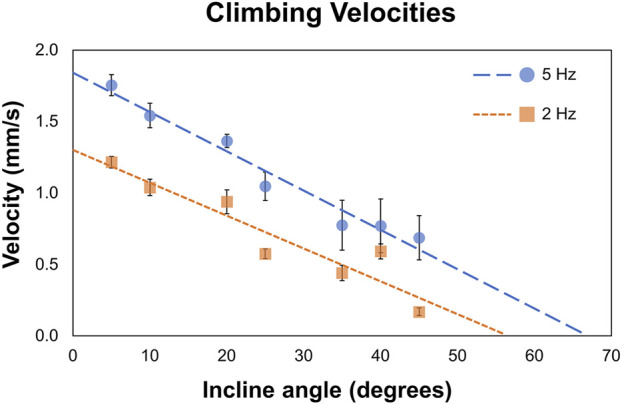
Climbing velocities. MANiACs climbing different inclines, from 5° to 45°, is shown at 2 and 5 Hz. Projections suggest that at 2 and 5 Hz the stall angle are approximately 55° and 66°, respectively.

We tested MANiACs on inclines of 5°, 10°, 20°, 25°, 35°, 40°, and 45° in aCSF and recorded average translational velocities ranging from 0.17 to 1.75 mm/s depending on both incline and rotation frequency. Figure 3 plots data for individual MANiACs climbing various slopes at 2 and 5 Hz (Figure 3). Our measurements provided evidence that MANiAC surface climbing follows the expected trends in that faster rotation rates induce faster climbing velocities and steeper inclines induce slower climbing velocities.

At 5 Hz, average climbing velocities of 0.77 ± 0.22 mm/s (35° incline) and 0.64 ± 0.17 mm/s (45° incline) indicate MANiACs were capable of climbing significant angles at clinically reasonable speeds. Surface tumbling translation methods allowed for linearly increasing translational velocities with increases in rotation frequency, granted sufficiently strong magnetic fields are applied so that the robot remains in phase with the applied rotating field (i.e., below the critical frequency). We observed that, under the magnetic conditions generated, MANiACs were not able to climb inclines with angles greater than 45°. These results are useful in understanding how velocity, rotation frequency, and incline angle will affect MANiAC motion. As CNS tissues such as the cortex are not flat surfaces, these climbing velocities have implications for how MANiACs may move on curved tissues during drug delivery.

### MANiACs Moving Against Flow

**FIGURE 4:**
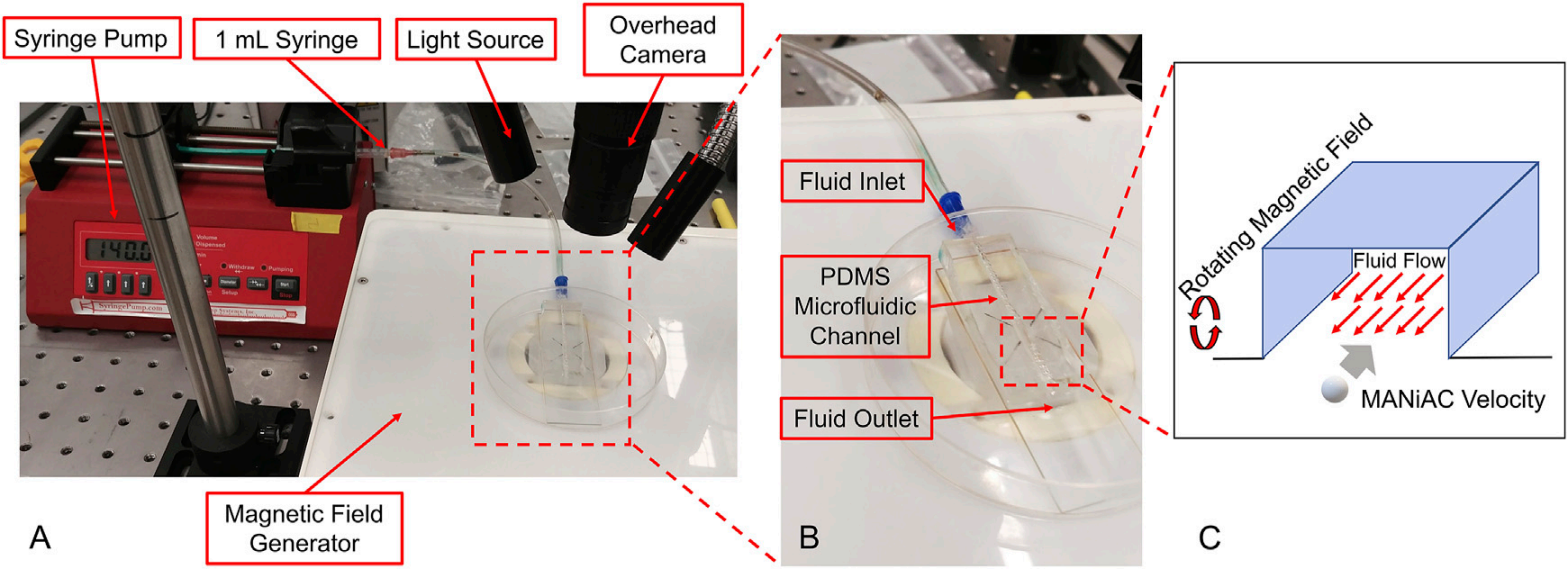
**(A and B)** Experimental setup for MANiACs motion under controlled flow rates. Using DI water as a fluid, rotation rates of 1, 3, and 5 Hz were tested against a range of flow rates from 0 to 200 ml/h or the maximum flow rate for which MANiACs were able to move against the flow. Videos of the motion were recorded for each flow rate and rotation rate pair, then the MANiAC's position over time were tracked using ImageJ. From this data, the velocities were calculated and averaged across experiments. Using blood as the fluid medium, the rotational frequency remained constant (5 Hz) throughout the experiments. **(C)** These experiments focused on MANIAC speed against different flow rates for different concentrations of K2EDTA treated whole blood (1, 5, and 10%) in PBS as a fluid medium.

**FIGURE 5:**
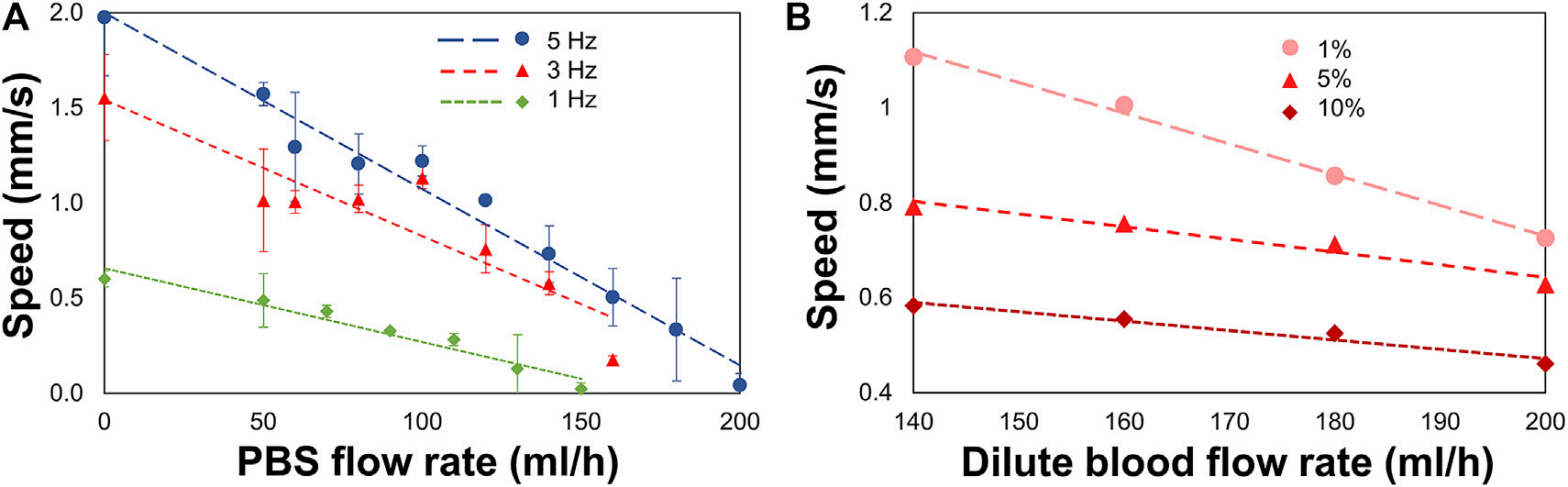
MANiAC speeds against flow rates in PBS and diluted K2EDTA-treated whole mouse blood. **(A)** MANiACs demonstrated translation against flow at low rotation frequencies (1–5 Hz). Lower rotation frequencies result in slightly lower stall flow rates (flow rate at which MANiAC velocity falls to nearly 0 mm/s). Rotation at 5 Hz enabled MANiACs to translate (move) against flow of 180 ml/h. **(B)** Translation speeds of MANiACs (5 Hz) in 1, 5, and 10% mouse blood. Interestingly, translation in mouse blood is less dependent on flow rate, possibly because blood constituents allow for increased coupling between the surface of the fluidic device and the MANiACs.

Tissues of the CNS are bathed in CSF that is constantly flowing. In order to understand and predict how magnetically actuated soft robotic drug delivery devices will move through flowing fluids and fluid-carrying vessels in the body, it is helpful to test such devices under similar flow conditions *in vitro*. Here we demonstrated magnetic rotation and surface tumbling of MANiAC translation against biologically relevant flow velocities. Under an imposed constant velocity flow, MANiACs were rotated and translated in a fluidic channel and tracked from above (Figure 4). Channels were 5 cm long, 2 mm wide, and 2 mm high, however imaging and tracking were performed along a 5-mm long segment of the channel. MANiACs were successfully moved against fluid flow in both PBS and diluted mouse blood. Higher rotation speeds resulted in fast translations in both PBS and diluted blood, and high fluid flow rates induced slower translation speeds for MANiACs (Figure 5). Importantly, MANiACs were able to translate against flow of up to 200 ml/h at 5 Hz in PBS and in 10% K2EDTA-treated whole mouse blood. Experimental results of the MANiAC motion against flow are shown in Figure 5.

Variability in translation velocities for a given flow rate condition was most likely due to size, shape, and magnetic nanorod loading differences among the MANiACs themselves. Other sources of variation included the separation distance and interaction dynamics of each capsule with the floor and sidewall surfaces of the fluidic device. Capsule to capsule variability in nanorod alignment, loading density and distribution, shape, and size all contributed to variability in transport dynamics in flowing fluid environments.

**FIGURE 6:**
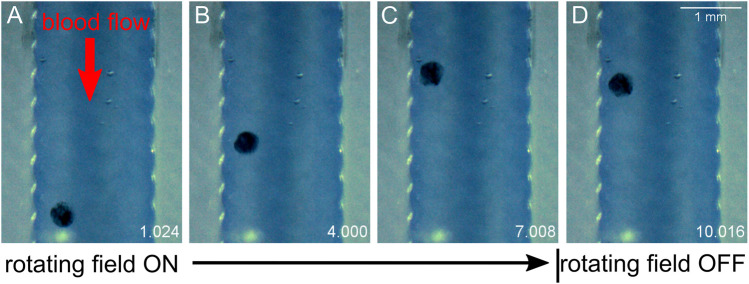
Translation against flow. MANiAC moving against 160 ml/h flow in 10% whole mouse blood. Time is listed in the lower right of each frame in units of seconds. **(A)** Applied 5 Hz rotating magnetic field is initiated, and **(B)** MANiAC begins to move against the flow of blood. **(C)** Rotating magnetic field is turned off and **(D)** MANiACs begins to move in the direction of blood flow. Video frames are separated by 3 s. Video included in Supplementary Information.

We view experiments in dilute blood as a good testing ground for translation experiments in fluids which involve proteins and cellular components. Dilute blood has a broader range of chemical and biological constituents as compared with buffer and aCSF. *In vivo*, CSF does carry proteins and cell fragments, and thus has some analogous fluidic characteristics to diluted mouse blood that is not entirely captured by aCSF. Additionally, there is a general interest in translation through moving blood in medical micro-robotics. As anticipated, higher concentrations of blood increased the viscosity of the fluid and decreased MANiAC transport speeds. In mixtures above 10% whole mouse blood, MANiACs were no longer visible from above due to the opacity of the mixture. Importantly, MANiACs were not dramatically hindered by the protein and cellular constituents in diluted mouse blood, nor did proteins or cell adsorption to the MANiACs cause immediate stalling of translation against blood flow. Figure 6 shows representative video frames of a MANiAC moving against flow in a PDMS channel. While rotating magnetic fields were turned on, the MANiAC moved against flow (Figures 6A–C), however upon turning off the rotating fields the MANiAC began to move with the induced flow (Figure 6D).

### Painting the Cortex

**FIGURE 7:**
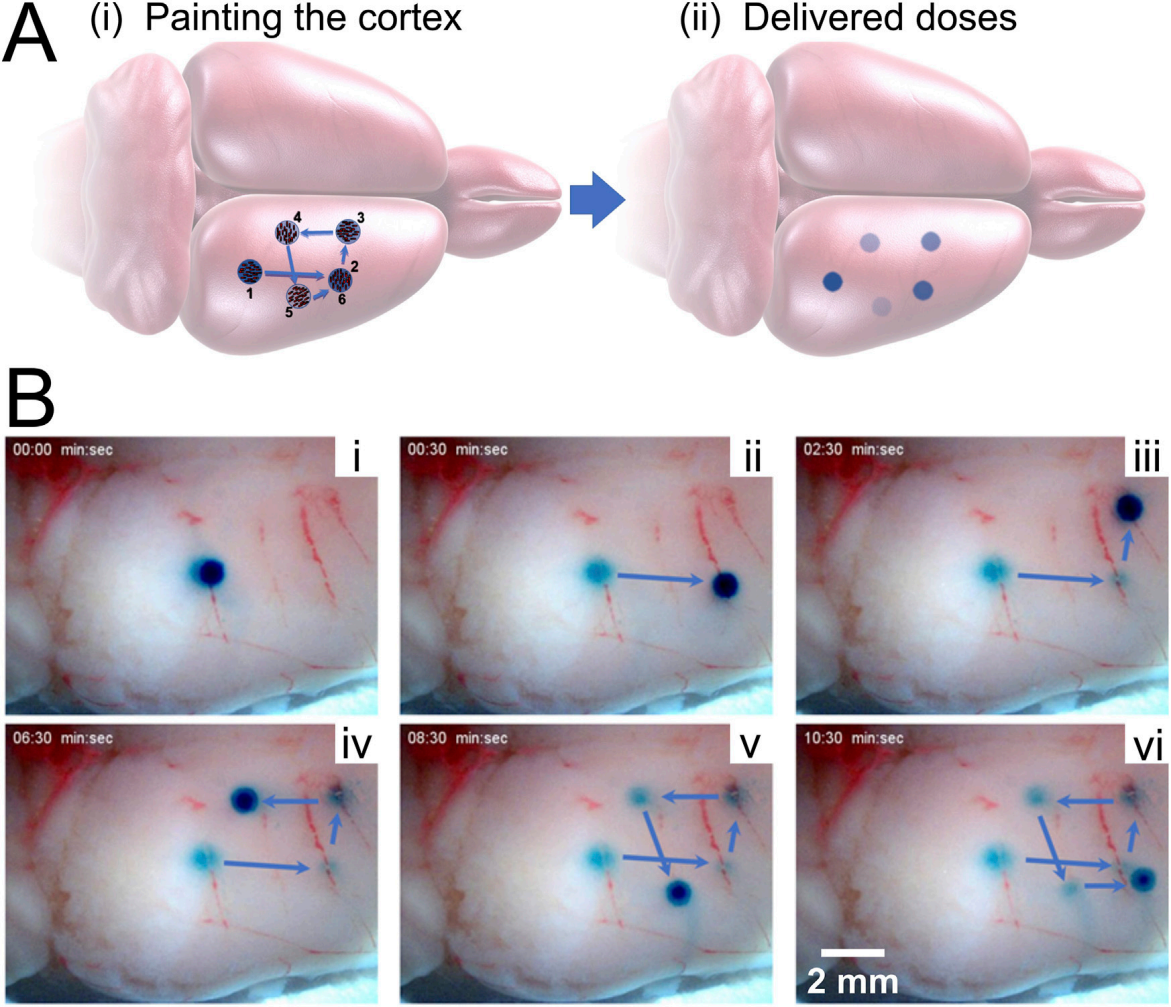
Painting the cortex. **(A)** Schematic showing the process of painting the rat cortex, showing target regions 1–5, with redosing (target 6) occurring at the same location as target region 2. **[(A), ii]** depicts the expected decrease in dose intensity as the dose experiment proceeds. **(B)** Experimental results showing painting a rat cortex *ex vivo*. **[(B), i**] through **[(B), v]** demonstrate the first five novel painting deliveries, while **[(B), vi]** demonstrates redosing. Only the right cortex is shown, with the MANiAC first moving in the anterior direction **[(B), i–ii]**, followed by translation in the medial direction **[(B), iii]** followed by motion in the posterior **[(B), iv]** lateral, **[(B), v]**, and finally anterior direction again. The entire process takes ∼10 min. Video included in Supplementary Information.

After loading with BG, MANiACs were larger than their pre-loaded dimensions as expected. After rinsing in DI water, they appeared as dark blue-green entities. BG-loaded MANiACs were placed on top of the rat occipital cortex at the border of the parietal cortex and then steered into location for cortex painting via BG release. Using tumble-and-pause manipulations, we target and release payload to various sites on the parietal cortex in aCSF. Figure 7 demonstrates the ability to target five different regions of the cortex with individual doses (Video included in Supplementary Material). Additionally, Figure 7 demonstrates the ability to redose (deliver payload multiple times) a specific target site (target site two in shown experiment). Redosing is an important capability as it demonstrates the ability to increase dose at a specific location even after an initial dose has been deposited. It is important to note that the BG dyed the cortical tissue, i.e., is released and then being absorbed by the tissue, as opposed to simply hovering in the solution around the MANiAC. Tissue treatment was evidenced by the fact that 1) cortex painting remained observable after thoroughly rinsing the tissue with both DI water and saline, and 2) the continued motion of the MANiACs did not result in a disturbance of the dyed area, as would occur for dye floating in solution above the tissue. During tumbling manipulations between target regions, some release of BG was observed. However, because transport between target regions only lasted a few seconds, the overall off-target release of BG into the surrounding aCSF and the tissue between target sites was small compared to the deposition at the dyed tissue region. This was apparent from the lack of connecting dose lines between target sites. Given the long time necessary for target site dosing (>1.5 min), translations over tissue that last ∼10 s was not expected to result in significant off-target dosing (Figure 7).

**FIGURE 8:**
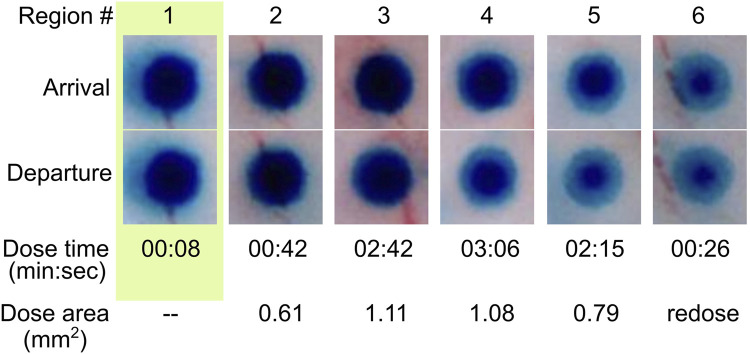
Time lapse release of BG dye from MANiAC. BG distribution in MANiACs before and after dosing six regions (labeled in top row). Optical images of MANiACs upon arrival at and departure from target regions 1–6. Shown is a MANiAC sitting on top of tissue releasing the BG at the six different dose sites, with a decreasing circumferential intensity as the BG is released from the perimeter of the capsule. The arrival row shows the MANiAC's color upon arriving at the dose site, and the departure row shows MANiAC's color just before the MANiAC leaves the dose site. Due to burst effect dynamics, the core of the MANiAC maintains high concentrations of BG after several doses and several minutes of BG release. BG depletion from the periphery of the MANiAC is optically evident by dose number 4. Time is in units of min:sec. Dose area is given as square millimeters.

There is no intrinsic limit for the minimum distance between doses aside from the diameter of the dosing MANiAC, as the rotation-based walking mechanism is fully continuous and induces translation distances which are directly proportional to the number of degrees of magnetically induced rotation. The upper limit for dose size is defined by the amount of payload which can be varied by an individual MANiAC. Close-up of BG release from capsules, along with dose times and measured dose areas (in mm^2^) for target regions is shown in Figure 8. It is important to observe that, due to the burst effect (Huang and Brazel, 2001) and the decreasing reservoir of BG in the MANiAC as time progresses, later target sites will require longer dose times to accomplish the same dose that is delivered during earlier times. We successfully demonstrated that, despite the curvature of the cortex, magnetic drug commandeering and targeting were possible over centimeter-length scales.

### Painting the Spinal Cord

The spinal cord, being surrounded by low viscosity CSF, offers an excellent opportunity for MANiAC-based targeted delivery. The spinal cord contains two significant grooves—the anterior median fissure and the posterior median sulcus. While the posterior median sulcus is rather narrow, the anterior median fissure is approximately 0.5 mm in diameter in rats and 3 mm in diameter in humans.

**FIGURE 9:**
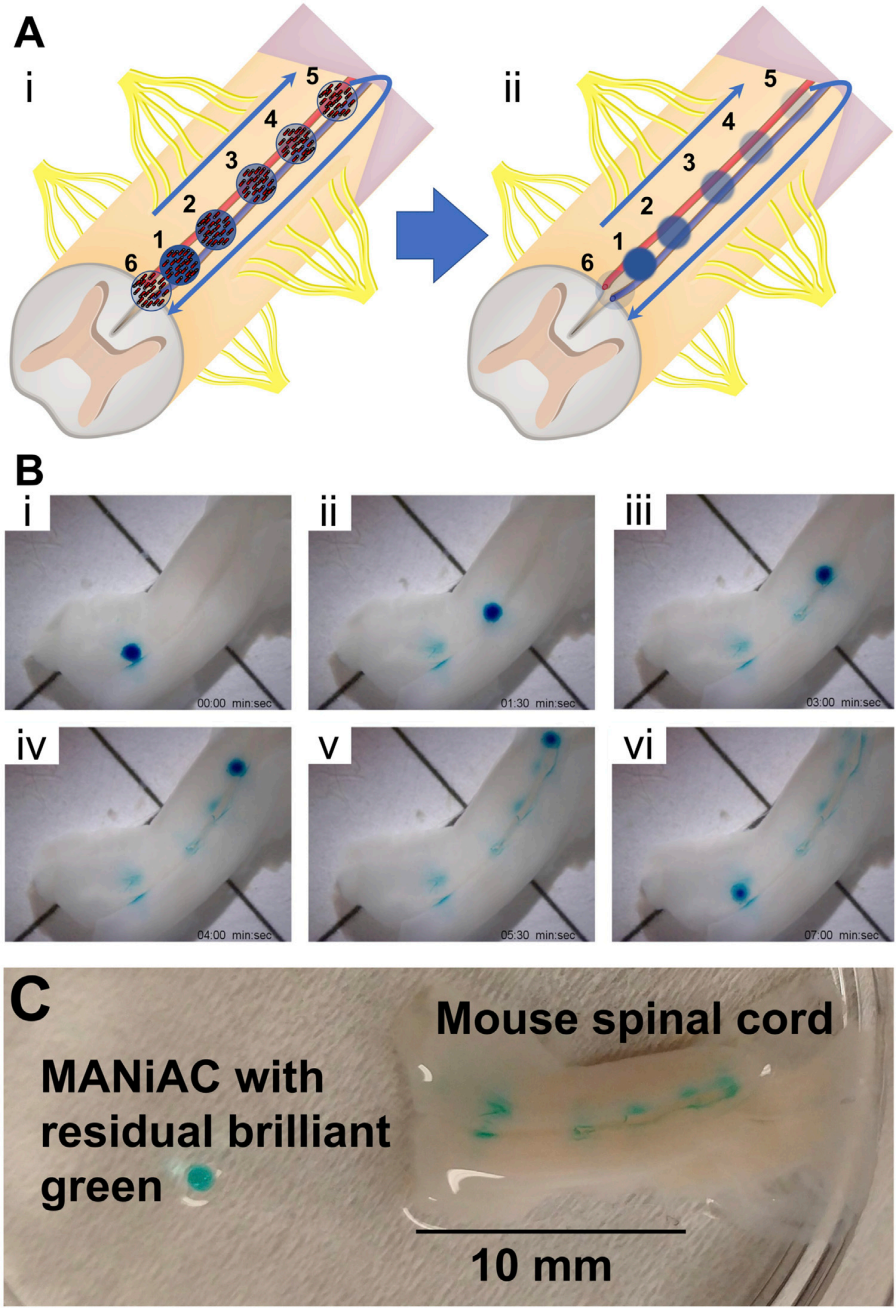
Painting the mouse spinal cord. **(A)** Schematic of painting the spinal cord depicts [**(A), i]** walking the MANiAC up and then back down the anterior median fissure of mouse spinal cord tissue, **[(A), ii]** painting the tissue in five locations. **(B)** Five locations along the spinal cord are painted with BG dye **[(B), i–v]** and the MANiAC is finally repositioned at the original starting point **[(B), vi]**. **(C)** Final painted mouse spinal cord with removed MANiAC having residual brilliant green dye. The entire procedure took ∼7 min. Video included in Supplementary Information.

We applied one BG-loaded MANiAC into the anterior median fissure of an excised mouse spinal cord and demonstrate delivery of BG payload to six target sites in aCSF. Over the course of 9 min the MANiAC was manipulated from the anterior end towards the posterior end, then back up to the anterior end, painting five distinct segments of the approximately 1.5 cm long mouse spinal cord. The final dose was a redosing of target site 1 at the end of the painting process (Figure 9, Video included as Supplementary Material). Importantly, unlike in experiments on the cortex, steering in the spinal cord fissure acted to contain the MANiAC within the fissure and deviations in steering angle with respect to the spinal cord were gently corrected by the walls of the fissure. As with painting the cortex, painting the spinal cord tissue was confirmed by demonstrating the BG was still in the tissue after rinsing the spinal cord in water and buffer (Figure 9C). Figure 9C also makes it clear that at target regions 2 and 3, dosing was slightly asymmetric in the lateral dimension, possibly due to asymmetric contact between the tissue and the capsule during those dosing events.

## Discussion

Staining the brain *via* accessing the CSF was first performed by E. E. Goldman in 1913, and those early studies significantly informed understanding of the blood brain barrier (BBB) (Goldman, 1913). Bypassing the BBB for delivery to CNS tissues directly via the CSF may enable a host of devices which would not pass through the BBB to be used as delivery tools (Naseri Kouzehgarani et al., 2021). As untethered milli- and microscale robotics progress towards applications in medicine, their mobility and metrics of control will need to be validated extensively. The capabilities and mechanisms of surface walkers have been demonstrated extensively *in vitro*, with detailed control over and interactions with mammalian cells, bacteria, microfabricated mazes, and small tissue samples. However, their ability to effectively explore large centimeter scale regions of critical CNS tissues has, to our knowledge, not been reported. As these tissues exhibit high levels of spatial-functional mapping, we envision surface walkers as a possible tool in the fight against various CNS diseases for which localized delivery may be highly beneficial. This may include targeting cancer with chemotherapeutic agents or applying small molecule drugs with high specificity in cases of neurodegenerative diseases or chronic pain. In the current study, multiple regions within the parietal cortex were targeted following initial application in the occipital lobe. The parietal cortex contains the primary somatosensory cortex, a structure with high spatial organization. Indeed, it has been suggested that reorganization of somatosensory and motor regions of the cortex following peripheral nerve injury may represent a target for the treatment of chronic pain (Li et al., 2021). The primary motor cortex adjacent to the somatosensory cortex in the frontal lobe is another potential target, although not tested in the current study. Another potential application related to these cortical regions is treatment following ischemic injury, such as stroke. Both sensory and motor deficits are often observed, and targeted treatment of these cortical regions could represent novel therapeutic modalities.

Rotational manipulation of millirobots is a particularly useful technique for treating diseases of the CNS as it enables fast motion of robots and relies on uniform rotating fields which can be applied at human scale distances (Rahmer et al., 2017). Future work aims to improve upon this work by including pulsatile fluid flow that more accurately characterizes CSF dynamics in the spinal cord and around the cortex, characterizing manipulations on CNS tissues *in vivo*, and painting CNS tissues with more relevant drugs and drug cocktails. It is anticipated that pulsatile flow conditions will add complexity and challenges to both capsule positioning control as well as capsule dosing dynamics. Additionally, a method for converting the demonstrated passive, diffusion-driven release dynamics to a controlled, triggered release dynamic is critical. Adding a coating to the MANiACs that allows for using radiofrequency heating of incorporated magnetic particles to induce coating opening and payload release may be used to enable temporal control over the drug delivery process. Combining improvements in payload release with advances in feedback control and real-time imaging will be required for experiments in animals and translation to humans.

While centimeter scale regions of the cortex could be targeted using our rotating propulsion method, the outer edge of the tissue was untreatable using rotational magnetic field control, as near the edge of the cortex MANiACs fell off the surface and were unable to climb back up onto the tissue. In such cases, MANiACs may be recovered by manipulation with a magnetic field gradient. Here we observe that MANiACs are able to access approximately 70% of the top surface of the rat cortex without falling off the surface. As has been shown previously, rotationally manipulated objects can climb steep inclines (Zhang et al., 2010). The ability to rotate at higher frequencies may enable MANiACs to effectively explore larger regions of the cortex while avoiding falling off the tissue. For a rat in a standard prone orientation, a MANiAC’s ability to treat the cortex depends on a balance of propulsive walking force with gravitational force at a given angle. At the stall angle, the propulsion force and gravitational force are equivalent such that

$$F_{∥}=6\pi \eta r^{2}\omega F_{∥}^{r*}-F_{g,∥}=0$$

where the coefficient $F_{∥}^{r*}$ describes the fluid coupling between rotational torque and translational force in the direction parallel to the cortex surface. Recognizing that the effective force of gravity in the parallel direction is given by $F_{g,∥}=4/3\pi r^{3}\left(\rho_{s}-\rho_{f}\right)g\sin\theta$ , and using $F_{∥}^{r*}≅-2/15\ln\left(\delta /r\right)\;$ in the limit of small (δ/r) (Goldman et al., 1967), then the stall angle can be calculated as

$$\sin\theta =\;-\frac{9}{15}\;\frac{\eta }{\left(\rho_{s}-\rho_{f}\right)g}\;\frac{\omega }{r}\ln\frac{\delta }{r}$$

where θ is the stall angle, η the viscosity of the fluid, and $\rho_{s}$ and $\rho_{f}$ the densities of the sphere and the fluid, respectively, r the radius of the sphere, g the acceleration of gravity, ω the rotation rate (in rad/s), and δ the separation distance between the brain tissue and the sphere. Preliminary assessment of the stall angle at frequencies 1–10 Hz suggest that MANiACs should experience stall angles between 1 and 10°, assuming fluid-mediated interactions between the surface and the capsule. As our climbing experiments on microfabricated inclines and CNS tissues demonstrate the ability to climb significantly steeper surfaces, we suggest an intermittent contact process that allows for capsule-surface interactions that, periodically, move outside the realm of entirely viscous-fluid-mediated interactions. As such, further improvements in translational velocities may be possible with careful engineering of magnetic forces, possibly including the addition of a magnetic gradient which acts to gently pull the capsules towards the surface during magnetically favorable times in a capsule’s rotation cycle (i.e., when the nanorods in the capsule are oriented within a few degrees of being perpendicular to the floor or surface).

In targeting the CNS, appropriate regions within the layers protecting the cortex and spinal cord must be targeted. Moving outwards, CNS tissues are protected by the pia mater, the arachnoid mater, and the dura mater, three layers of increasingly dense, tough tissues which protect the CNS cells from mechanical damage, as well as biological and chemical invasion. Here, capsules, placed directly on top of the cortex and the spinal cord were sufficient to dose tissues repeatedly, and to generate doses sufficient to withstand rinsing after experiments were completed. This is likely aided by the high solubility and small size of BG, making it easy to absorb. BG has a molecular weight of 482.64 Da, which is not significantly smaller than some relevant drugs used for treating CNS disease, such as methotrexate (MW ≈ 508 Da). Future experiments will combine such relevant drugs and cocktails combinations of therapeutics for demonstrating multi-therapeutic simultaneous delivery.

Because the pia mater is permeable to water and small solutes, insertion and transport of MANiACs through the subarachnoid space between the arachnoid mater and pia mater may allow for delivery of small molecules to the brain and spinal cord *via* transport through the pia mater. Detailed knowledge of the rate of release will allow for highly quantitative and tuned release profiles at specific locations along the cortex or spinal cord. Translational studies will also need to take into account patient-specific information, including the size of the subarachnoid space (SAS) as determined by diagnostic imaging, so as to ensure particle transport is not hindered due to extremely narrow SAS dimensions. Smaller MANiACs may need to be developed for specific populations or disease states in which the SAS is particularly narrow.

While numerous reports of nanoparticle-generated fluid flows have been reported (Nguyen, 2012), rotational manipulation of surface walkers against directed flow have been more challenging to demonstrate. Magnetic nanoparticles in simple fluids have been moved at impressive speeds against flow (Karle et al., 2010). However soft millirobots translating against flow in complex blood solutions has, to our knowledge, not been demonstrated. CSF flow rates vary widely across the CNS. Patients diagnosed with ALS have been measured to have CSF flow rates of 0 ml/h along the entire spinal canal (Sass et al., 2020), while healthy patients have been estimated to have daily net flow rates of 295 ml/h at the cerebral aqueduct and 4,500 ml/h at the cranio-cervical junction (Lindstrøm et al., 2018). CSF flow is inherently pulsatile, with instantaneous flow rates on the order of 1–2 ml/s in both cranial and caudal directions along the human spine (Dreha-Kulaczewski et al., 2018). As such, manipulations of MANiACs for delivery to cranially directed sites in the spine may rely on magnetic pulses that hold the capsule in place during caudally directed CSF flow, then apply rotational manipulation during low velocity and cranially directed CSF flow. To address the complex flows and contours of the CSF, it is clear that innovative methods for manipulating soft robotic devices will need to be devised.

## 5 Conclusion

As milli- and micrometer scale devices advance towards payload delivery to specific tissues, the ability to commandeer MANiACs to surface walk on a tissue offers one route to treating a particular region with highly targeted, concentrated doses of small molecules or other payloads. Alginate capsules loaded with magnetic nanorods can climb 45° inclines, move against fluid flow in buffer and diluted blood, and can be loaded with a small-molecule payload for manipulation on CNS tissues from rats and mice. We have demonstrated a simple, straightforward method for controlled, targeted delivery of payload to the accessible surfaces of the cortex and the spinal cord using MANiACs manipulated by rotating magnetic fields.

## Data Availability

The raw data supporting the conclusions of this article will be made available by the authors, without undue reservation.
